# Exploring the impact of the care sport connector in the Netherlands

**DOI:** 10.1186/s12889-017-4830-6

**Published:** 2017-10-16

**Authors:** Karlijn E. F. Leenaars, Eva Smit, Annemarie Wagemakers, Gerard R. M. Molleman, Maria A. Koelen

**Affiliations:** 1 Department of Social Sciences, Health and Society Group, Wageningen University & Research Centre, P.O. Box 8130, 6700 EW Wageningen, The Netherlands; 2 Academic Collaborative Centre AMPHI, Primary and Community Care, Radboud university medical center, P.O. Box 9101, 6500 HB Nijmegen, The Netherlands

**Keywords:** Intersectoral collaboration, Broker role, Physical activity promotion, Primary care, PA sector

## Abstract

**Background:**

Regular physical activity (PA) is deemed to contribute to the primary and secondary prevention of several chronic diseases, like diabetes mellitus, cancer, cardiovascular diseases, and osteoporosis. In 2012, Care Sport Connectors (CSC), to whom a broker has been ascribed, were introduced in the Netherlands to stimulate PA and guide primary care patients towards local sport facilities. The aim of this study was to explore which structural embedding is the most promising for CSCs’ work.

**Methods:**

In three rounds of interviews, 13 CSCs were followed for 2 years in their work. In these interviews, a network survey was used to identify organisations in the CSCs’ network, whether they collaborated with these organisations, and the role of the organisations in the connection. Data from the network survey were analysed using the RE-AIM framework and disaggregated into how CSCs were structurally embedded (Type A: only PA sector; Type B: different sectors; Type C: partnership). A related samples Wilcoxon signed rank test was performed to study how the CSCs’ network developed between 2014 and 2016.

**Results:**

All CSCs established a connection between the primary care and the PA sector in which the average number of organisations with which CSCs collaborated increased significantly between 2014 (8.3) and 2016 (19.8) (*p* = 0.002). However, differences were identified in the way CSCs were structurally embedded and in the way they established the connection. Type A CSCs established the connection mostly around their own activities, supported PA organisations with their activities, and collaborated with primary care and welfare professionals around their own activities. Type B and Type C CSCs established the connection by organising, supporting, and implementing different kinds of activities targeting different kinds of audiences, and collaborated mostly with primary care professionals around the referral of professionals’ patients.

**Conclusions:**

The results of this study suggest that adopting an integral approach (Type B and C) for the structural embedding of the CSC is more promising for reaching the desired outcomes. Whether CSCs really improve the target groups’ PA level and health needs to be further studied.

**Trial registration:**

Dutch Trial Register NTR4986. Registered 14 December 2014.

**Electronic supplementary material:**

The online version of this article (10.1186/s12889-017-4830-6) contains supplementary material, which is available to authorized users.

## Background

Regular physical activity (PA) is associated with enhanced health and reduced risk of all-cause mortality, and has many health benefits [1]. Therefore, regular PA is deemed to contribute to the primary and secondary prevention of several chronic diseases, like diabetes mellitus, cancer, cardiovascular diseases, and osteoporosis [2]. About 40% of Dutch adults do not meet the Dutch recommendation about being moderately active for 30 min at least 5 days per week [3].

In order to stimulate PA, in 2012 the Dutch Ministry of Health, Welfare, and Sport introduced neighbourhood sport coaches (*Buurtsportcoach),* ascribing to them a broker role. These coaches are 40% funded by the state and 60% funded by the municipality or other local organisations. Some of these coaches, called Care Sport Connectors (CSCs), are employed specifically to connect the primary care sector (all care that is directly accessible to the patient, i.e. GP, physiotherapist, dietician) and the PA sector (all PA services in the neighbourhood, i.e. sport clubs, fitness centres, PA lessons at community centres, and walking groups) in order to guide primary care patients towards local PA facilities. A blueprint for the implementation of CSC funding or function was not prescribed, allowing municipalities to implement CSCs in line with local needs and contexts. The general idea is that CSCs facilitate collaboration between professionals in the primary care and PA sector; that activities to promote PA are implemented; and reach target groups that need to be more physically active. The overall aim is that target groups that need to be more physically active are reached and health outcomes will improve.

It is desirable to connect the primary care and the PA sector because of the potential for reaching physically inactive adults [4]. Primary care professionals are in an ideal position to motivate their patients to be physically active, and the PA sector has a range of PA activities. However, previous studies have shown that differences between both sectors (different cultures and interests) [5–7] and barriers relating to their own sector (primary care professionals’ lack of time and knowledge, and lack of suitable PA activities) [8–12] can hinder their mutual collaboration. A broker role holds the promise of improving intersectoral collaboration [13].

Although a broker role seems promising for improving intersectoral collaboration, to our knowledge no study has yet evaluated the impact of a broker role on connecting both sectors. In our review study, which described collaborative initiatives between the primary care and the PA sector, we found one initiative [14] that made use of a broker to organise a partnership of community organisations to promote PA [15]. Although that study showed that, according to professionals, the broker role was effective in carrying out their work, the study focused on the results of the partnership for PA promotion rather than on the broker role for improving intersectoral collaboration [14]. The CSC function provides an excellent opportunity to study the impact of the broker role on improving intersectoral collaboration.

A previous study, which explored the CSC role, revealed that the role is promising for improving collaboration between the primary care and the PA sector. However, the way in which municipalities structurally embedded CSCs influenced the ease with which CSCs could initiate collaboration structures [16]. For example, CSCs working from the PA sector found it harder than CSCs working from other organisations to involve primary care professionals. Therefore, the aim of this study is to try to explore which structural embedding is the most promising for CSCs’ work. The research questions addressed are: 1) how does the CSCs’ network develop over time, 2) how do CSCs establish the connection between the primary care and the PA sector, and 3) what is the impact of structural embedding of CSCs on CSCs’ work?

## Method

### Study design

A multiple case study was conducted from 2014 to the end of 2016 in nine municipalities spread over the Netherlands. To analyse the CSCs’ network, the connection established and the impact of structural embedding on CSCs’ work, 13 CSCs were, in three rounds of interviews, followed for 2 years in their work.

### Setting and study population

The nine municipalities were selected through convenience sampling based on project partners’ contacts. The main inclusion criterion was: municipalities implementing the CSC function for 4 years (until 2017). During the selection, we made sure that municipalities of different sizes and from different regions of the Netherlands were included (≤ 300,000 inhabitants (*n* = 2), 100,000–300,000 inhabitants (*n* = 4), ≤ 100,000 inhabitants (*n* = 3)). In these municipalities, the CSC was structurally embedded differently. In four municipalities, CSCs (*n* = 5) were structurally embedded in the PA sector only (Type A). In the other five municipalities, an integral approach (involving multiple organisations and sectors) was adopted to structurally embed CSCs. Two forms of this integral approach could be distinguished: four CSCs were working from primary care, or welfare, or PA organisations (Type B), and four other CSCs were part of a partnership between primary care, welfare, and PA organisations (Type C).

In consultation with the representative policymaker in each municipality, CSCs were selected to participate in this study. Inclusion criteria were: the CSC 1) aims to connect the primary care and the PA sector and 2) is working with an adult target group, preferable adults who could benefit from PA. The selected 13 CSCs represent approximately 15% of the CSCs employed to connect primary care and sport for adults in the Netherlands.

The average age of the 13 CSCs (4 men, 9 women) was 33 years (min 27 years, max 57 years). Ten CSCs had a bachelor’s degree, two had a master’s degree, and one had a vocational education diploma. At the time of the first interview in 2014, six CSCs had been in position for 0–6 months, four CSCs had been working for 6–12 months, and three for longer than a year. Type A CSCs worked on average 26.4 h, type B CSCs 28.5 h, and type CSCs 27.5 h (Table 1).

**Table 1:** Study participants and the connection established between both sectors by the study participants

	Municipality	CSC	Connection established between the primary care and the PA sector
1.	• Number of inhabitants: 100,000–300,000 • Structural embedding Type A: PA sector	1. • Personal: woman, 52 years, higher education, municipal sport department • In position: > 1 year • Number of hours: 20	• CSC activity: organisation of fitness test and guiding participants towards PA facilities, organisation of different PA activities • Referral: sporadic • Supporting organisations: guiding participants towards PA facilities • Supporting CSC: provision of rooms, spreading information
2.	• Number of inhabitants: ≤ 100,000 • Structural embedding Type A: PA sector	2. • Personal: woman, 57 years, community college, municipal sport department • In position: 6–12 months • Number of hours: 16 • Addition: stopped temporarily due to illness in 2014–2015	• CSC activity: organisation of fitness test, organisation of different PA activities • Referral: sporadic • Supporting organisations: guiding participants towards PA facilities • Supporting CSC: provision of rooms, spreading information
3.	• Number of inhabitants: 100,000–300,000 • Structural embedding Type A: PA sector	3. • Personal: woman, 28 years, higher education, municipal sport department • In position: 6–12 months • Number of hours: 24	• CSC activity: organisation of fitness test, organisation of new PA activities • Referral: sporadic • Supporting organisations: guiding participants towards PA facilities • Supporting CSC: provision of rooms, spreading information
4.	• Number of inhabitants: ≤ 100, 000 • Structural embedding Type A: PA sector	4. • Personal: man, 27 years, higher education, sport organisation • In position: 6–12 months • Number of hours: 36	• CSC activity: organisation of fitness test, organisation of PA activities • Referral: sporadic • Supporting organisations: guiding participants towards PA facilities • Supporting CSC: spreading information
		5. • Personal: woman, 30 years, university, sport organisation • In position: 6–12 months • Number of hours: 36	• CSC activity: organisation of fitness test, organisation of PA activities • Referral: sporadic • Supporting organisations: guiding participants towards PA facilities, education for sport clubs • Supporting CSC: spreading information
5.	• Number of inhabitants: ≥ 300,000 • Structural embedding Type B: Care, welfare, and sport organisation	6. • Personal: woman, 29 years, higher education, sport organisation • In position: 0–6 months • Number of hours: 32	• CSC activity: organisation of fitness test, organisation of PA activities • Referral: structural referral programme • Supporting organisations: guiding participants/primary care patients towards PA facilities; supporting PA instructors, sport clubs, and community health centre to create new activities • Supporting CSC: spreading information, provision of rooms
6.	• Number of inhabitants: 100,000–300,000 • Structural embedding Type B: Care, welfare, and sport organisation	7. • Personal: woman, 27 years, higher education, sport organisation • In position: 0–6 months • Number of hours: 32	• CSC activity: organisation of PA activities • Referral: regularly • Supporting organisations: guiding primary care patients/residents towards PA facilities, supporting sports clubs to reach new members, supporting community health centre with their PA activity • Supporting CSC: network meetings, spreading information
		8. • Personal: woman, 31 years, higher education, welfare organisation • In position: 0–6 months • Number of hours: 20	• CSC activity: organisation of new PA activity • Referral: structural referral programme. • Supporting organisations: guiding primary care patients towards PA facilities • Supporting CSC: network meetings, spreading information
7.	• Number of inhabitants: 100,000–300,000 • Structural embedding Type B: Care, welfare, and sport organisation	9. • Personal: woman, 55 years, university, health broker at the Municipality Health Service • In position: > 1 year • Number of hours: 30	• CSC activity: networking meeting • Referral: not present • Supporting organisations: supporting health and welfare professionals (for example integral fall prevention programme) • Supporting CSC: spreading information
8.	• Number of inhabitants: ≥ 300,000 • Structural embedding Type C: Partnerships between primary care, welfare, and PA professionals	10. • Personal: man, 30 years, higher education, welfare organisation • In position: 0–6 months • Number of hours: 30	• CSC activity: inventing, organising and carrying out PA activities, partnership meeting • Referral: structural consultation hour at community health centre • Supporting organisations: guiding primary care patients/residents towards PA lessons at community centre • Supporting CSC: spreading information, introduction to other organisations, develop joint plan
		11. • Personal: man, 45 years, higher education, care organisation • In position: 0–6 months • Number of hours: 36	• CSC activity: inventing, organising and carrying out PA activities, partnership meeting • Referral: regularly • Supporting organisations: guiding primary care patients/residents towards PA facilities, supporting existing PA programmes • Supporting CSC: spreading information, introduction to other organisations, developing joint plan
		12. • Personal: man, 35 years, higher education, welfare organisation • In position: 0–6 months • Number of hours: 24	• CSC activity: consultation hour at primary care organisations, partnership meeting • Referral: structural referral scheme • Supporting organisations: supporting PA instructors • Supporting CSC: spreading information, introduction to other organisations, developing joint plan
9.	• Number of inhabitants: ≤ 100,000 • Structural embedding Type C: Partnerships between primary care, welfare, and PA professionals	13. • Personal: woman, 47 years, higher education, community health centre • In position: > 1 year • Number of hours: 20	• CSC activity: variety of PA programmes for primary care patients • Referral: structural referral program • Supporting organisations: organisation of PA activities in collaboration with PA clubs, guiding primary care patients towards PA facilities • Supporting CSC: partnership meeting, developing joint plan

*CSC* Care Sport Connector

*PA* physical activity

### Data collection

To analyse the CSCs’ network, the connection established and the impact of the structural embedding on CSCs’ work, both quantitative and qualitative data were collected. Quantitative data on the CSCs’ network was collected using Frey et al.’s [17] Levels of Collaboration Survey. With this survey, we identified organisations in the CSCs’ network, their role, and the level of collaboration (network – cooperation – coordination – coalition – collaboration) on a scale ranging from 1 to 5. The different levels of collaboration, characterised as described in Frey et al. [17], are presented in Additional file 1. During the first interview round, it appeared that CSCs found it hard to distinguish the different collaboration levels and often chose only network (an organisation with which they had contact) or collaboration (an organisation with which they collaborated). Therefore, in the second and third interview rounds, we used the Levels of Collaboration Survey only to identify organisations in the CSCs’ network, whether they collaborated with these organisations, and the manner in which they collaborated.

The assessment of the CSCs’ network was completed during three rounds of interviews, each with a time span of approximately 6 to 12 months (March–May 2014, March–May 2015, March–May 2016). Qualitative data were therefore also collected during the three rounds, in which CSCs further explained their scores on Frey et al.’s Levels of Collaboration Survey. In one case, an interview did not take place in 2015, because the CSC temporarily ceased functioning. Therefore, the number of CSCs in 2015 was 12. The interviews took place at the CSCs’ workplace and lasted between 1 and 1.5 h. The interview rounds were performed by KL and ES.

### Data analysis

To analyse CSCs’ network, the connection established and the impact of structural embedding on CSCs’ work, Glasgow et al.’s RE-AIM framework [18] was used. The RE-AIM framework conceptualises the public health impact of an intervention as a function of five factors: reach, efficacy, adoption, implementation, and maintenance. Normally, this framework is used to evaluate intervention impact on individual behaviour change, but the RE-AIM framework has been deemed feasible to evaluate broad, multi-faceted initiatives that incorporate multiple interventions targeted at a variety of audiences [19, 20]. CSCs work with different kinds of professionals to implement different kinds of activities targeting different kinds of audiences (e.g. professionals and target group) in order to promote PA [16]. In our opinion the RE-AIM framework is therefore also suitable for this study.

To apply the RE-AIM framework in the CSC context, operationalisations of the factors were adopted based on the operationalisations as described in Sweet et al. [19] and Finch et al. [20].

- Reach: refers to the (average) number of organisations in the CSCs’ network with which they actually had contact, but with which they did not work.
- Efficacy: refers to the CSCs’ main objective and the result of the connection between both sectors [18]: increased level of PA among the target group. Data collection on groups addressed by CSCs and CSCs’ impact on stimulating PA and the health among these groups is still going and therefore efficacy could not be addressed at this moment.
- Adoption: refers to the (average) number of organisations in the CSCs’ network with which they collaborated.
- Implementation: refers normally to the extent to which a programme was implemented as intended. However, a blueprint for implementation was not provided, only that the various sectors had to be connected. Therefore, implementation here refers to how CSCs established that connection. Four forms of collaboration were identified in the CSCs’ network: collaboration around a specific CSC activity, a referral scheme, CSCs’ support to an organisation (for example guiding residents or primary care patients towards PA facilities), or organisations supporting CSCs in their work (for example introducing new partners to CSCs).
- Maintenance: refers to the extent to which CSC sustained collaboration with organisations over the years.

The factors reach, adoption, implementation, and maintenance were calculated from the quantitative data collected from the network survey.. For each CSC network, the number of organisations was counted to provide an answer on each factor of the RE-AIM framework. Subsequently, descriptive statistics were used to calculate means for the total group and the subgroups (Types A, B, and C) for each factor of the RE-AIM framework for each year.

To study how the CSCs’ network developed between 2014 and 2016 (organisations reached by CSCs (reach) and organisations with which CSCs collaborated (adoption), a related samples Wilcoxon signed rank test was performed, because the data for reach and adoption were not normally distributed.

To study how CSCs establish a connection between the primary care and the PA sector, the implementation factor of the RE-AIM framework was used. Calculated averages were computed to illustrate which forms of collaborations were established in the CSCs’ network and the kind of professionals involved in the forms of collaboration. The assessment of the CSCs’ network took place during an interview in which more detailed information was collected on how the connection between both sectors was established. This information allowed us to describe the factor implementation more specifically.

To study the impact of structural embedding on CSCs’ work, calculated averages of the subgroups were computed to illustrate differences between the form of structural embedding and CSCs’ work. The groups were too small (*n* = 4) to perform statistical analyses to compare the RE-AIM framework factors between the forms of structural embedding.

## Results

### Reach

The average number of organisations in the CSCs’ network increased significantly over the years (p.002) (Table 2). In 2014, CSCs reached on average 12.9 (SD = 5.8) organisations; in 2016, this was 24.5 (SD = 7.1).All CSC networks consisted of primary care, PA, welfare, and other organisations such as schools, representatives of municipalities, and existing partnerships. Primary care organisations were the most present in the CSC networks over the years (Table 3).

**Table 2:** Development of Care Sport Connectors’ network over time

	2014		2015		2016			
	*M*	*SD*	*M*	*SD*	*M*	*SD*	*Z* ^a^	*P*
Reach	12.9	5.8	20.8	5.9	24.5	7.1	−3113	.002
Adoption	8.3	4.1	14.1	3.9	19.8	5.5	−3112	.002

^a^Z statistics obtained from a related samples Wilcoxon signed rank test between 2014 and 2016

**Table 3:** Care Sport Connectors’ impact on connecting the primary care and the physical activity sector

	Type A: PA sector (*n* = 5, 2015 *n* = 4)			Type B: Different sectors (*n* = 4)			Type C: Partnership (*n* = 4)		
	2014	2015	2016	2014	2015	2016	2014	2015	2016
Reach^a^	12.2 (SD = 7.1)	22 (SD = 5.4)	22.4 (SD = 6.8)	14.3 (SD = 5.7)	21.3 (SD = 3.0)	27.3 (SD = 4.6)	12.5 (SD = 5.5)	19.3 (SD = 9.4)	24.3 (10.2)
Primary care	4.6	8.5	9.6	3.8	7	9.5	4.3	5.3	7.3
PA sector	3.4	7.3	5.6	4	5.5	6.5	2.8	4.8	5
Welfare	2.2	3.8	2.8	2	3.3	3.5	1.3	4	4.5
Others	2	3.5	4.4	4.5	5.3	7.8	4.3	5.3	7.5
Adoption^a^	7.6 (SD = 5.0)	15.3 (SD = 3.2)	18.2 (SD = 4.9)	10.5 (SD = 4.8)	16.3 (SD = 3.5)	24.5 (SD = 5.4)	7 (SD = 0.8)	10.8 (SD = 3.1)	17.3 (SD = 4.0)
Primary care	3.4	5.3	8.6	2.5	6.3	9	2.8	3.3	6.5
PA sector	2.2	6.8	5	2.5	3.3	6.3	0.8	2	2.8
Welfare	0.6	1.3	1.6	1.8	2.5	3.3	1.3	2.3	3.8
Others	1.4	2	3	3.8	4.3	6	2.3	3.3	4.3
Implementation^a^									
Collaboration around activity CSC	3.2	5.5	8.6	1.3	1.5	4.3	0.5	1.8	4.5
Collaboration around referral	1.6	2	2.4	1.3	5	6.5	1.5	3	5
CSC supporting other organisations	3	7	7.4	5.5	7	10.5	0.3	2.5	3.8
Other organisations supporting CSC	0.6	2	2.4	4.3	5.3	6	5.3	6.3	6.8

^a^Average number of organisations

*CSC* Care Sport Connector

*PA* physical activity

No major differences were found between the CSCs’ reach and type of structural embedding (Type A: 22.4, Type B: 27.3, Type C: 24.3). However, minor differences could be seen in the structure of CSCs’ networks and type of structural embedding. Type A CSCs’ networks consisted mostly of primary care and PA professionals, whereas the networks of Type B and Type C were more diverse and consisted also of welfare professionals and other organisations such as municipalities and schools (Table 3).

### Adoption

The average number of organisations with which CSCs collaborated increased significantly over the years (p.002) (Table 2). In 2014, CSCs collaborated on average with 8.3 (SD = 4.1) organisations; in 2016, this was 19.8 (SD = 5.5).Over the years, CSCs collaborated mostly with primary care professionals (Table 3).

Type B CSCs collaborated with more organisations than the other CSCs. Especially in 2016, the average number of organisations with which Type B CSCs collaborated was larger (24.5, min = 21, max = 32) than that of Type A CSCs (18.2, min = 12 max = 23) and Type C CSCs (17.3, min = 13, max = 22). Differences could also be found in the structure of the CSCs’ network and type of structural embedding. Type A CSCs collaborated mostly with primary care and PA organisations over the years, whereas the other CSCs collaborated with a different range of organisations: primary care, PA, welfare, and other organisations such as schools, community centres, and existing partnerships.

### Implementation

**Fig. 1:**
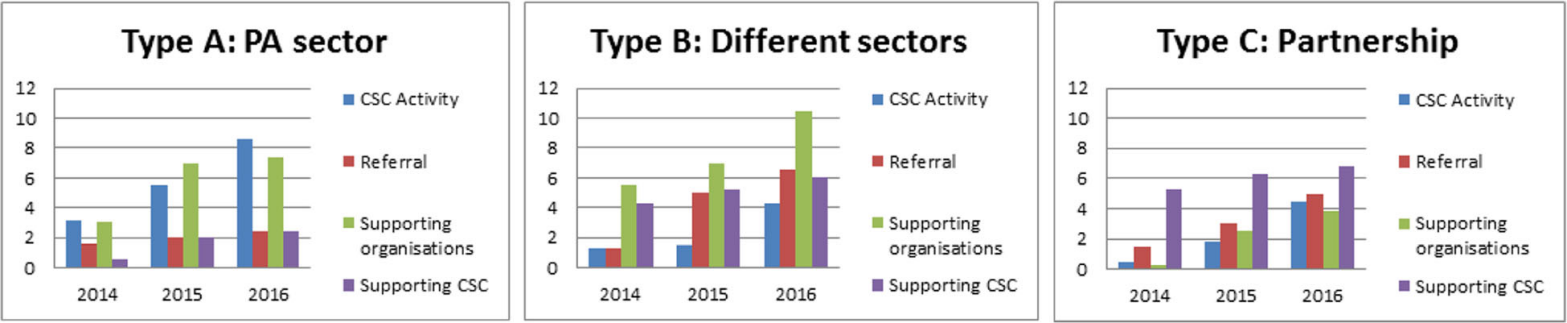
Average number of organisations per form of collaboration disaggregated into the structural embedding of the CSC

**Fig. 2:**
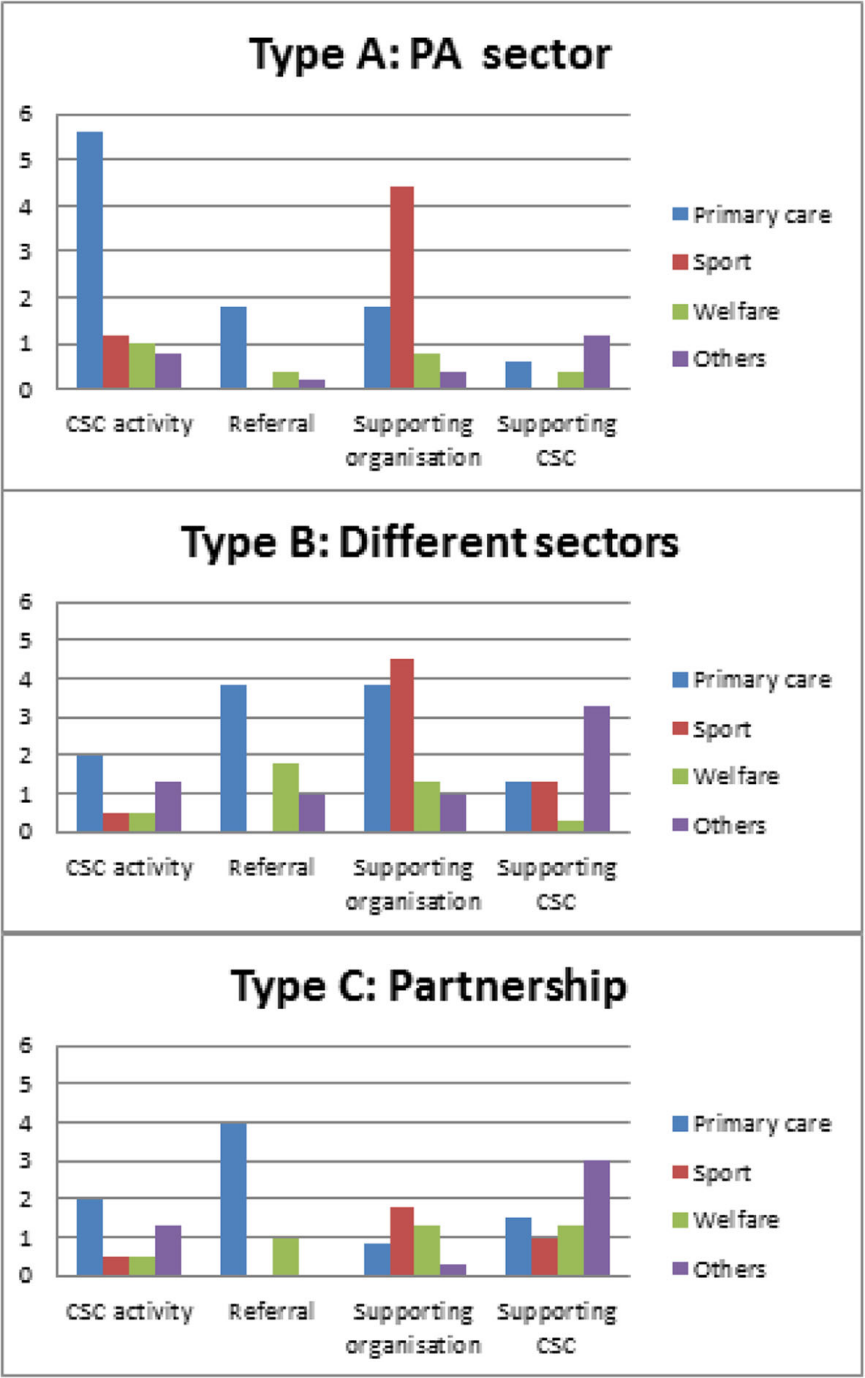
Average number of type of organisations per form of collaboration disaggregated into the structural embedding of the CSC

During the study period, all CSCs established a connection between the primary care and the PA sector. Table 1 provides detailed information on the connection established. Differences could be distinguished in how the connection between both sectors was established and the type of structural embedding (Figs. 1 and 2, and Table 3).

Type A CSCs established a connection between both sectors mostly by organising fitness tests to reach residents and guide them towards local PA facilities (Table 1). Type A CSCs collaborated thus mostly with other organisations around their own activities (8.6, min = 5, max = 12) or supported organisations with their activities (7.4, min = 5, max = 10). To a lesser extent, these CSCs collaborated with primary care and welfare professionals around the referral of the target group (2.4, min = 0, max = 8) and with organisations that could support CSCs in their work (2.4, min = 0, max = 4).

Types B and C CSCs established the connection between both sectors in different ways. CSCs organised their own PA activities, implemented a structural referral scheme, supported PA and primary care organisations, or organised network meetings (Table 1). They thus collaborated with the professionals around all identified activities: their own activities (Type B: 4.3, min = 1, max = 7; Type C: 4.5 min = 3, max = 7), referral (Type B: 6.5, min = 0, max = 18; Type C: 5, min = 2, max = 8), supported organisations with their activities (Type B: 10.5, min = 1, max = 19; Type C: 3.8, min = 1, max = 7), and had professionals in their network who supported the CSCs with their work (Type B: 6, min = 4, max = 8; Type C: 6.8, min = 1, max = 11).

The difference in professionals’ role in the connection established and the type of structural embedding related to the way the connection between both sectors was established (Fig. 2). Type A CSCs collaborated mostly with primary care and welfare organisations around their own activities (6.6, min = 3, max = 10) and supported mostly PA organisations with their activities (4.4, min = 1, max = 7). The other CSCs collaborated mostly with primary care and welfare organisations around the referral of their patients (Type B: 5.6, min = 0, max = 15; Type C: 5, min = 2, max = 8) and supported primary care, welfare, and PA professionals with their activities (Table 3).

### Maintenance

The CSCs sustained collaboration with organisations in their network over the years. CSCs collaborated in 2014 with an average of 8.3 organisations (Table 3) and sustained this collaboration with an average of 6.2 organisations (min = 1, max = 13) in 2016. During the 2 years of this study, an average of 4.1 organisations (min = 0, max = 7) stopped their collaboration with the CSC.

Minor differences could be found between the type of structural embedding and organisations that sustained their collaboration. Type A CSCs collaborated in 2014 with an average of 7.6 organisations and sustained this collaboration with an average of 5 organisations (min = 1, max = 13) in 2016. Type B collaborated in 2014 with an average of 10.5 organisations, with which CSCs sustained collaboration with an average of 7.8 organisations (min = 3, max = 13) in 2016. Type C collaborated in 2014 with an average of 7 organisation and sustained this collaboration with an average of 5.8 organisations (min = 5, max = 7) in 2016.

## Discussion

In this study, we analysed CSCs’ network and the connection established in order to explore which structural embedding is the most promising for CSCs’ work in connecting the primary care and the PA sector and guiding primary care patients towards local PA facilities. All types of CSCs had organised a similar network of reached organisations and established a connection between both sectors. The results of this study show that a structural embedding guided by an integral approach seems the most promising for CSCs’ work, although no major differences were found between the two forms of this integral approach (Types B and C CSCs). This structural embedding is the most promising because it is related to: 1) the way the connection between both sectors was established and 2) the role of primary care professionals in the connection.

First, Type A CSCs established the connection between both sectors mostly around their own activities to promote PA and supported mostly PA organisations by guiding residents towards their activities. Types B and C CSCs established the connection between both sectors by organising, supporting, and implementing different kinds of activities targeting different kinds of audiences – for example, a structural referral scheme, network meetings, and supporting primary care and PA organisations with their activities. It is plausible that municipalities that adopt an integral approach to structurally embed the CSC create a greater impact, because of these different activities targeting different audiences. A minor difference between the two types of integral approach was noticed. Type B CSCs collaborated on average with more organisations and sustained this collaboration with more organisations than Type C CSCs did. An explanation for this difference is that the Type C CSCs collaborated mostly with the organisations within their partnership.

Secondly, Types B and C CSCs collaborated mostly with primary care professionals around the referral of primary care patients, whereas Type A CSCs mostly collaborated with these professionals around their own activities in which primary care professionals were involved with the implementation of CSCs’ activities. Because of the different roles of primary care professionals, probably a different kind of target group (primary care patients vs. general population) will be reached by CSCs. This is especially true because the connection between the primary care and the PA sector can be characterised as multidisciplinary [21].The connection can mostly be seen as a chain in which CSCs guide the target group towards PA facilities after referral by primary care professionals or their own recruitment. The role of primary care professionals in the referral of their patients is therefore important for reaching target groups who could benefit from PA. Preliminary results of a study that is part of the larger project on reaching target groups indicated that residents reached by CSCs themselves scored better on several health outcomes than residents referred by primary care and welfare professionals towards CSCs did.

The differences in impact and type of structural embedding can be understood by the context in which CSCs are working. In a previous study in which we explored CSCs’ operational context, it appeared that municipalities that adopted an integral health and PA policy and an embedding of this policy in partnerships at management level also used an integral approach to structurally embed the CSC [22]. These CSCs are thus working in municipalities in which collaboration between different sectors is part of their policy and embedded in other municipal operations, such as the implementation of health and PA promotion programmes by different organisations. In addition, the adoption of an integral approach to structurally embed the CSC created support for the connection among primary care, welfare, and PA organisations. For those CSCs, establishing collaboration, especially with primary care and welfare organisations, was easier than for CSCs working only from the PA sector [16].

In our study, we used Frey et al.’s Level of Collaboration Survey [17]. However, it appeared in the first interview round that it was very hard for CSCs to identify the differences between the levels, and they often chose one of the extremes. Therefore, in the second and third interview rounds, the scale was not used and changes in the levels were not studied. Nevertheless, the descriptions of the different levels of collaboration provided in all interviews meant that the CSCs described their form of collaboration with the professionals very precisely. Therefore, conducting the Level of Collaboration survey during an interview helped us to gain a full understanding of the way collaboration between both sectors was established - especially because at first sight the CSCs organised similar networks. In addition, to explore CSCs’ impact on connecting the primary and the PA sector, we espoused the RE-AIM framework. Although normally this framework is used to evaluate intervention impact on individual behaviour change, it appeared that the RE-AIM framework was suitable and useful for analysing the data from the network analysis and for studying the impact of an intervention on intersectoral collaboration. Other studies have experienced the same potential of the RE-AIM framework to move beyond evaluation of single interventions or settings and to study the impact of multi-faceted real-life initiatives that incorporate multiple interventions targeted to a variety of audiences [19, 20]. In line with these studies, we adopted a more pragmatic approach to the RE-AIM framework, focussing on utilising the strengths of different quantitative and qualitative methods to evaluate comprehensively the impact of CSCs’ work. Therefore, the RE-AIM factors were operationalised accordingly. Unfortunately, proportions of the reach and adoption (percentages of organisations reached by/collaborated with and could be reached by/collaborated with CSCs) could not be calculated because of the absence of information on of potential partners for CSCs. However, as it appeared that all types of CSCs had a similar network, it was more interesting to know was, as studied under the implementation factor, how CSCs established the connection between both sectors and the role of the organisations in this connection.

As far as we know, this is the first study to explore the impact of a broker role on connecting the primary care and the PA sector. Therefore, a first insight on this topic is presented in this study. This insight is relevant for policymakers, municipalities, and organisations working on connecting the primary care and the PA sector. The results of this study imply that a blueprint to instruct municipalities to adopt an integral approach to structurally embed CSCs may be necessary to successfully connect both sectors and to reach the desired outcomes. Other studies, part of the larger project, need to reveal the impact of the CSC on stimulating residents’ PA and whether using an integral approach to implement the CSC funding is indeed the most promising way to promote PA among the target group of primary care patients.

### Study’s strength and limitations

By following 13 CSCs in their work for 2 years, we gained an in-depth insight into the CSCs’ impact on connecting the primary care and the PA sector; this is valuable for further studies on CSCs’ impact. However, some limitations need to be taken into account when these results are being interpreted. In order to explore CSCs’ impact on establishing a connection between both sectors, we used self-reported results. CSCs were asked during the interviews to elaborate on their network partners and their role. It is possible that the CSCs did not give a complete overview of their network or that they were overly optimistic about their established connection and the role of the organisations in this connection. However, networks were checked with the CSCs, and they could provide additional information after the interviews were conducted.

This study was conducted among a small population of CSCs, making it hard to formulate firm conclusions about the impact of the CSC on improving intersectoral collaboration. However, at the start of this project, not much was known of the CSC function and therefore an in-depth insight and an exploration of the CSC role and the way CSCs’ establish the connection between both sectors is more valuable and necessary at this time. In addition, because of the small population and as a result small subgroups, statistical analysis to compare the types of structural embedding was not possible. Further studies should study examine whether an integral approach is more promising in reaching the desired outcomes.

## Conclusion

This study explored CSCs’ impact on connecting the primary care and the PA sector. In addition, we explored the impact of structural embedding on CSCs’ work. Although all CSCs established a connection between both sectors, differences in impact were found between CSCs structurally embedded in the PA sector and CSCs structurally embedded according to an integral approach. The results of this study suggest that using an integral approach to structurally embed the CSC is more promising for reaching the desired outcomes. Whether CSCs can really reach the desired target group and improve the target group’s PA level and health needs to be further studied.

## Additional file

Additional file 1: Network survey used in this study. (DOCX 16 kb)

## Abbreviations

CSC: Care Sport Connector
PA: physical activity
