# Supplementary material for: Exploring the impact of the care sport connector in the Netherlands
Source: BMC Public Health. 2017 Oct 16;17:813. doi: 10.1186/s12889-017-4830-6 (PMC5644127; doi:10.1186/s12889-017-4830-6)
Supplement: Additional file 1: — Network survey used in this study. (DOCX 16 kb) [file 12889_2017_4830_MOESM1_ESM.docx]

**SUPPLEMENTARY FILE**

**Appendix A.1 Network survey used in this study**

|  |  |  | **Levels of collaboration and their characteristics *** | | | | |
| --- | --- | --- | --- | --- | --- | --- | --- |
| **Name of organisation with which the CSC has contact** | **Sector** | **Role in the connection**  *(contact – collaboration, form of collaboration)* | **Networking**   - *Aware of organisation* - *Loosely defined roles* - *Little communication* - *All decisions are made independently* | **Cooperation**   - *Provide information to one another* - *Somewhat defined roles* - *Formal communication* - *All decisions are made independently* | **Coordination**   - *Share information and resources* - *Defined roles* - *Frequent communication* - *Some shared decision making* | **Coalition**   - *Share ideas* - *Share resources* - *Frequent and prioritised communication* - *All members have a vote in decision making* | **Collaboration**   - *Members belong to one system* - *Frequent communication is characterised by mutual trust* - *Consensus is reached on all decisions* |
| 1. |  |  |  |  |  |  |  |
| 2. |  |  |  |  |  |  |  |
| 3. |  |  |  |  |  |  |  |
| 4. |  |  |  |  |  |  |  |
| 5. |  |  |  |  |  |  |  |

**In the second and third interview rounds, changes in level of collaboration were not part of the survey*
